# Supplementary material for: Morphological and Proteomic Analyses of Soybean Seedling Interaction Mechanism Affected by Fiber Crosslinked with Zinc-Oxide Nanoparticles
Source: Int J Mol Sci. 2022 Jul 3;23(13):7415. doi: 10.3390/ijms23137415 (PMC9266555; doi:10.3390/ijms23137415)
Supplement: Supplementary file 1 [file ijms-23-07415-s001.zip › Supplemental Figures.pdf]

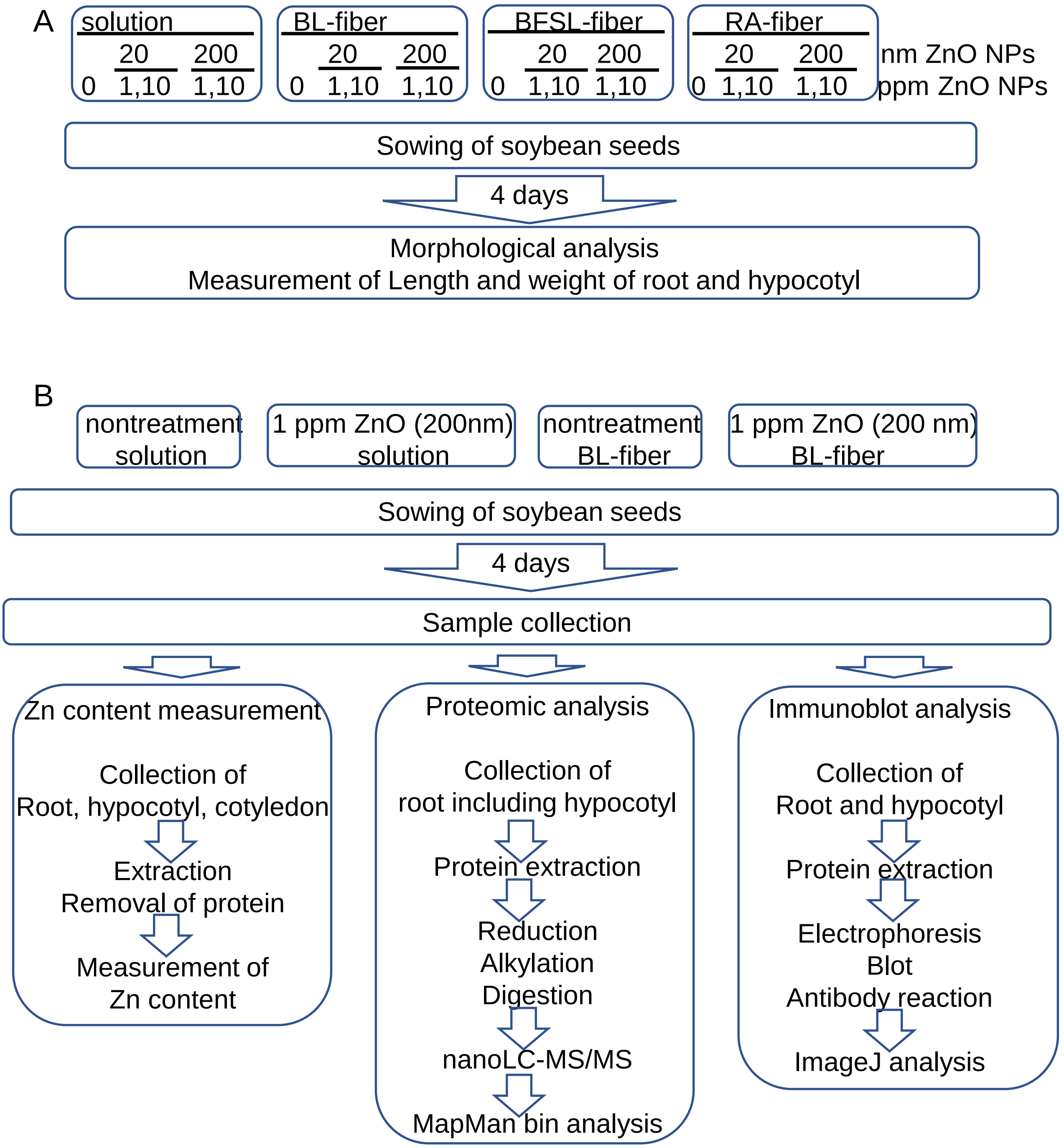

Figure S1. Experimental layout to study the effects of ZnO NPs on soybean. A) Morphological analysis. Soybean seeds were sown using solution, BL-fiber, BFSL-fiber, and RA-fiber with or without 1 and 10 ppm ZnO NPs (20 and 200 nm). B) Zn content measurement, proteomic analysis, and immunoblot analysis. Based on the result of morphological analysis, more effective conditions were selected. All experiments were performed with biological triplicates.

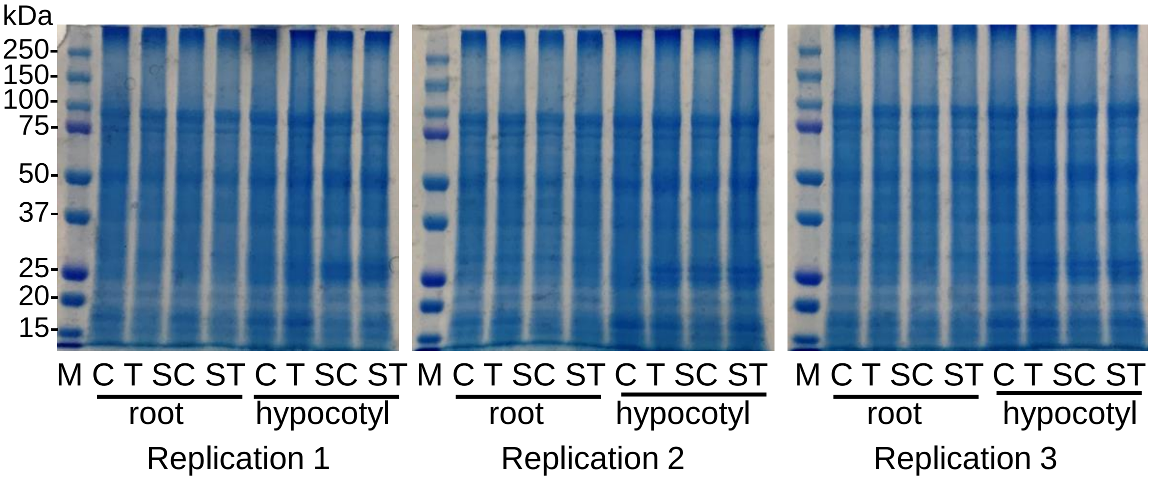

Figure S2. Coomassie brilliant blue pattern of biological triplicates. Proteins were extracted from the root and hypocotyl of soybean with or without treatment, separated on SDS-polyacrylamide gel and stained with Coomassie brilliant blue. M means marker proteins. C, T, SC, and ST mean nontreatment/ solution, 1 ppm ZnO NPs (200nm)/ solution, nontreatment/ BL-fiber, and 1 ppm ZnO NPs (200 nm)/ SE60G-fiber, respectively.

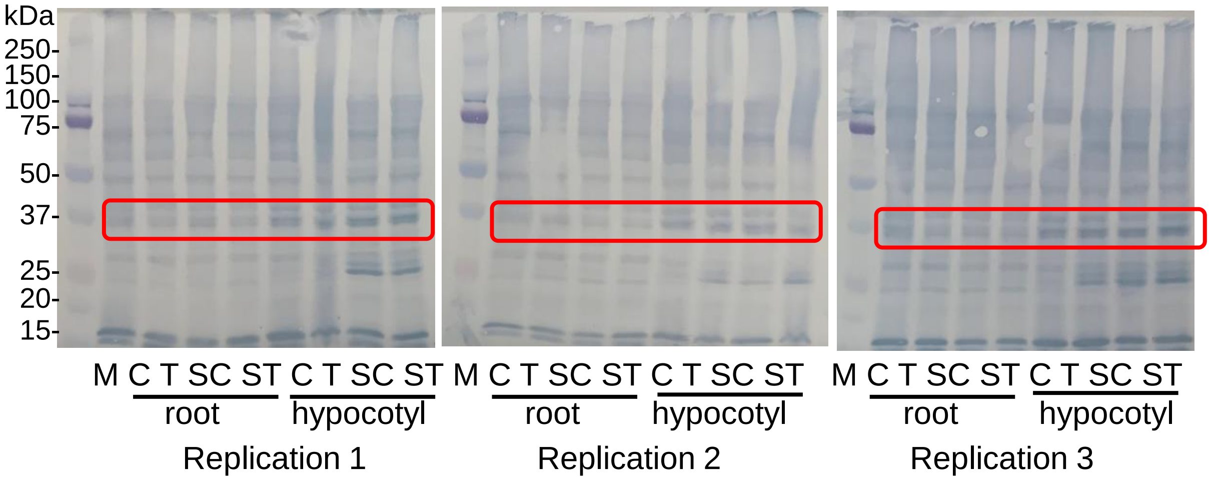

Figure S3. Three biological replicate data of immunoblot analysis using anti-NADPH oxidoreductase antibody for Figure 5A. Proteins were extracted from the root and hypocotyl of soybean treated with ZnO NPs, separated on SDS-polyacrylamide gel and transferred onto membranes. The membranes were cross-reacted with anti-NADPH oxidoreductase antibody. Coomassie brilliant blue staining pattern was used as a loading control. M means marker proteins. C, T, SC, and ST mean nontreatment/ solution, 1 ppm ZnO NPs (200nm)/ solution, nontreatment/ BL-fiber, and 1 ppm ZnO NPs (200 nm)/ BL-fiber, respectively.

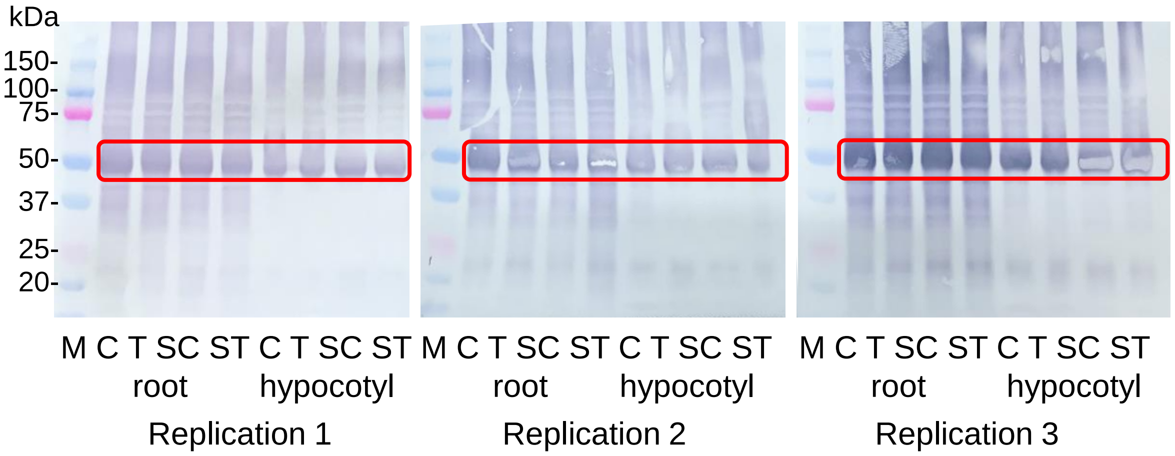

Figure S4. Three biological replicate data of immunoblot analysis using anti-alpha tubulin antibody for Figure 5B. Proteins were extracted from the root and hypocotyl of soybean treated with ZnO NPs, separated on SDS-polyacrylamide gel and transferred onto membranes. The membranes were cross-reacted with anti-alpha tubulin antibody. Coomassie brilliant blue staining pattern was used as a loading control. M means marker proteins. C, T, SC, and ST mean nontreatment/ solution, 1 ppm ZnO NPs (200nm)/ solution, nontreatment/ BL-fiber, and 1 ppm ZnO NPs (200 nm)/ SE60G-fiber, respectively.

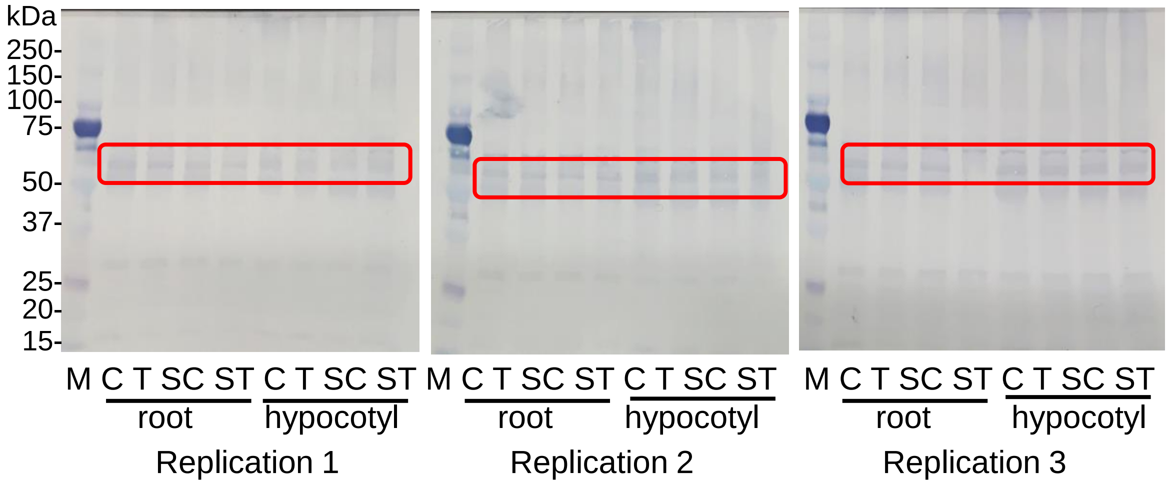

Figure S5. Three biological replicate data of immunoblot analysis using anti-beta tubulin antibody for Figure 5C. Proteins were extracted from the root and hypocotyl of soybean treated with ZnO NPs, separated on SDS-polyacrylamide gel and transferred onto membranes. The membranes were cross-reacted with anti-beta tubulin antibody. Coomassie brilliant blue staining pattern was used as a loading control. M means marker proteins. C, T, SC, and ST mean nontreatment/ solution, 1 ppm ZnO NPs (200nm)/ solution, nontreatment/ BL-fiber, and 1 ppm ZnO NPs (200 nm)/ SE60G-fiber, respectively.
